# Methyl-Donors Can Induce Apoptosis and Attenuate Both the Akt and the Erk1/2 Mediated Proliferation Pathways in Breast and Lung Cancer Cell Lines

**DOI:** 10.3390/ijms22073598

**Published:** 2021-03-30

**Authors:** Eva Kiss, Gertrud Forika, Reka Mohacsi, Zsuzsanna Nemeth, Tibor Krenacs, Magdolna Dank

**Affiliations:** 1 1st Department of Internal Medicine and Oncology, Oncology Profile, Semmelweis University, 1085 Budapest, Hungary; kiss.eva3@med.semmelweis-univ.hu (E.K.); mohacsi.reka@med.semmelweis-univ.hu (R.M.); titkarsag.dank@med.semmelweis-univ.hu (M.D.); 2 1st Department of Pathology and Experimental Cancer Research, Semmelweis University, 1085 Budapest, Hungary; forika.gertrud@med.semmelweis-univ.hu (G.F.); krenacs.tibor@med.semmelweis-univ.hu (T.K.)

**Keywords:** breast cancer, lung cancer, methyl-donors, apoptosis, complementary therapy

## Abstract

Dietary methyl-donors play important roles in physiological processes catalyzed by B vitamins as coenzymes, and are used for complementary support in oncotherapy. Our hypothesis was that methyl-donors can not only assist in tolerating cancer treatment but may also directly interfere with tumor growth and proliferation. Therefore, we investigated the proposed cancer inhibitory effects of methyl-donors (in a mixture of L-methionine, choline chloride, folic acid, and vitamin B12) on MCF7 and T47D breast cancer as well as A549 and H1650 lung cancer cell lines. Indeed, methyl-donor treatment significantly reduced the proliferation in all cell lines, possibly through the downregulation of MAPK/ERK and AKT signaling. These were accompanied by the upregulation of the pro-apoptotic Bak and Bax, both in MCF7 and H1650 cells, at reduced anti-apoptotic Mcl-1 and Bcl-2 levels in MCF7 and H1650 cells, respectively. The treatment-induced downregulation of p-p53(Thr55) was likely to contribute to protecting the nuclear localization and apoptosis inducing functions of p53. The presented features are known to improve the sensitivity of cancer therapy. Therefore, these data support the hypothesis, i.e., that methyl-donors may promote apoptotic signaling by protecting p53 functions through downregulating both the MAPK/ERK and the AKT pathways both in breast and lung adenocarcinoma cell lines. Our results can emphasize the importance and benefits of the appropriate dietary supports in cancer treatments. However, further studies are required to confirm these effects without any adverse outcome in clinical settings.

## 1. Introduction

Complementary care including proper diet, psychological support, and physical activity plays a greater role in the success of cancer treatment than considered before [1,2,3,4,5,6,7]. Elevated dietary methyl-donor intake, for instance, has been suggested to contribute to cancer prevention by reducing the risk of breast [8,9,10], lung [11], and colorectal cancers [12,13]. However, the mechanisms behind this has not been fully clarified, which attracted our attention to study the effects of methyl-donors in cancer.

Dietary methyl-donors, such as folate, betaine, choline, methionine, and other B vitamins such as B2, B6, and B12 in the food play important physiological roles, including amino- and nucleic acid metabolism, redox defense, cell growth, and apoptosis [1]. These components provide methyl groups for the one-carbon metabolism, including the methionine and folate cycles, besides being involved in the trans-sulfuration methylation pathways [2]. Inadequate DNA methylation may lead to the development of cancer, mainly by the shortage of the necessary vitamins and minerals for the proper functioning of these pathways [3]. Indeed, a recently published meta-analysis revealed that folate is associated with decreased risk of all-cause mortality and a wide range of chronic disease [14]. Another review reported similar results, where low or deficient folate status was associated with several cancers, except prostate cancer, which prevalence was linked with folic acid (FA) supplementation and a higher serum level [15]. The tight interrelationship between choline, methionine, and folate has already been known from in vivo studies, which recommend the testing of all three agents, when studying diet and DNA methylation [16]. The folate-mediated one-carbon metabolism (FOCM) is highly sensitive to nutrition status of several B vitamins as well, that alter network outputs [17]. Additionally, a majority of clinical and randomized clinical trials have shown that methyl-donor micronutrient intake affects DNA methylation by reducing the risk of several cancer types [3]. Furthermore, folate can induce apoptosis via the PTEN/AKT/P53 signaling pathway, and through reducing the effects of both the AKT and ERK signaling in breast cancer [18,19]. It is known that AKT signaling can inhibit the activation of the pro-apoptotic Caspase-9 and Caspase-3 [20], and that upstream inhibitors of the MAPK pathway can reduce tumor proliferation and mediate apoptosis, though Erk1/2 activation may also be involved in apoptosis induction [21]. Interestingly, a DNA methylation reader, methyl-CpG-binding protein 2 (MeCP2), regulates genes linked to tumorigenesis as well [22], and promotes proliferation via activation of Erk1/2, and its loss of function induces apoptosis [23]. Natural compounds and green tea polyphenols are able to downregulate MeCP2 [24,25]. B vitamins are also required for the energy-yielding metabolism, oxygen transport, and neuronal functions. Therefore, they are essential in cognitive and psychological functions, including mental and physical fatigue [5,6].

Breast cancer incidence is still growing worldwide, although the 5-year relative survival rate improved around 10% over the last 40 years, where prevention and early diagnosis contributed to almost half of this reduction even in breast cancer mortality [7]. Lung cancers account for the most cancer-related deaths worldwide [26]. The larger proportion of these cases are non-small cell lung cancers (NSCLC), which the majority are at advanced stages when diagnosed, thus they frequently develop resistance to the first- or second-generation-targeted therapies [26].

Cancer treatments primarily focus on the elimination or at least the reduction of tumor burden and on preventing cancer spreading to distant locations from the primary sites. However, almost equally important is to keep up both the mental and physical energy and motivation of the patients, which are required for their life activities as close to normal as possible, despite the trauma they are going through. To achieve this, it is important to support the sensitivity and reduce the side effects of cancer treatments, for their optimized efficiency. Methyl-donors, such as folate and cobalamin (B12) support of pemetrexed-based chemotherapy treated lung cancer patients, or pyridoxine (B6) and B12 as well as thiamin (B1), has only been applied in the recent treatment regimens to reduce the symptoms of the side effects induced by systemic chemotherapy [27,28,29]. Evidence-based complementary therapies, including dietary support, could greatly assist in reaching both of the above-mentioned targets [6,30].

In this work, we aimed to explore how methyl-donor treatments affect the growth, proliferation, apoptosis, and the related pathways in hormone positive invasive breast cancer (MCF7 and T47D) [31] and NSCLC lung cancer (A549 and H1650) cell lines.

## 2. Results

### 2.1. Methyl-Donors Affect Tumor Cell Proliferation

We used the MTS proliferation assay to detect the effects of methyl-donor treatments on cancer cell growth both in breast cancer and lung cancer cell lines. The highest concentration of methyl donors significantly decreased the proliferation rate in all cell lines (48 h and 72 h at MCF7 *p* < 0.05 and *p* < 0.01, respectively, 24 h at A549 *p* < 0.01, 72 h at T47D *p* < 0.01, and 72 h at H1650 *p* < 0.001) compared to the non-treated control.

### 2.2. The Effects of the Methyl-Donors on the Cell Cycle

**Figure 1:**
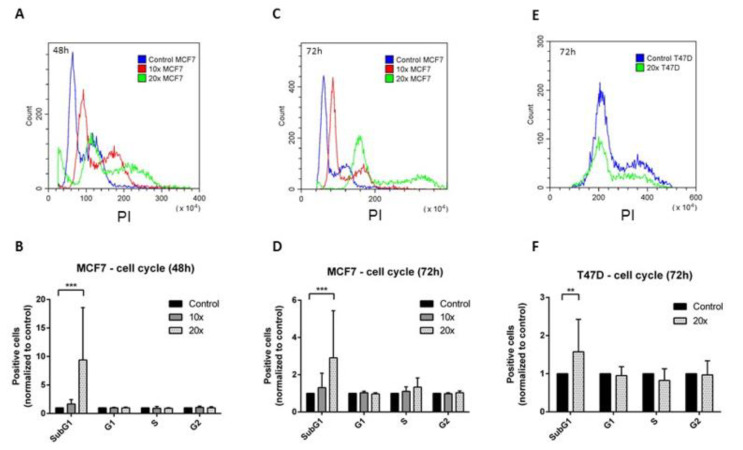
Cell cycle analysis of the MCF7 and T47D cells. SubG1 fractions of breast cancer cell lines were significantly increased after methyl-donor treatments compared to untreated controls. SubG1 fraction increased significantly in MCF7 cells both after 48 h (**A**,**B**) and 72 h (**C**,**D**), and in T47D cells after 72 h (**E**,**F**) treatments. Each bar represents the average number of positive cells normalized to control from at least 3 repeats ± SD. Statistical significance: **: *p* < 0.01 in T47D; ***: *p* < 0.001 in MCF7 cells. ×10 and ×20: concentrations of methyl-donors.

**Figure 2:**
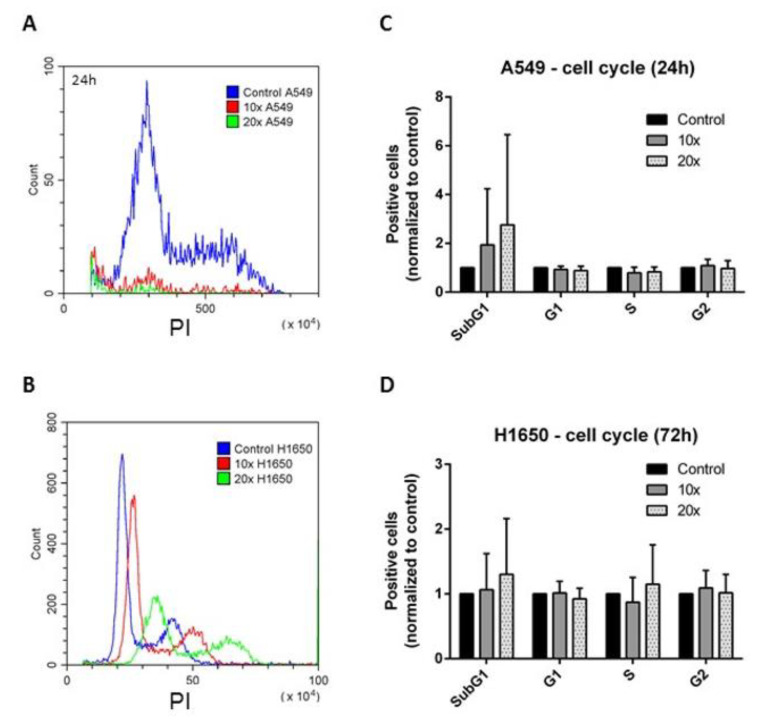
Cell cycle analysis of A549 and H1650 lung cancer cells. (**A**). Results of the cell cycle analysis of A549 by flow cytometry. (**B**). Results of the cell cycle analysis of H1650 by flow cytometry. C. Results of the statistical analysis of the cell cycle measurements of A549 cells (*n* = 4). D. Results of the statistical analysis of the cell cycle measurements of H1650 cells (*n* = 5). Only a tendency of increased SubG1 fractions were seen in A549 cells after 24 h (**A**,**C**) and in H1650 cells after 72 h (**B**,**D**) treatments (*p* = 0.35 and *p* = 0.46, respectively). ×10 and ×20: concentrations of methyl-donors.

Treatment related changes in the cell cycle and apoptosis were tested using flow cytometry. The subG1 fractions, indicating also the apoptotic cells, were increased in all methyl-donor treated cell lines. The changes were significant only in the breast cancer (*p* < 0.001 at both timepoints in MCF7, *p* < 0.01 in T47D cells) (Figure 1A–F), but not in the A549 and H1650 cell lines (Figure 2A–D). However, the inverse tendency of changes in the subG1 vs. G1 phase fractions, i.e., increase vs. decrease, respectively, were seen in all cell lines.

### 2.3. Detection of Apoptosis and Related Pathway Elements after Methyl-Donor Treatments

**Figure 3:**
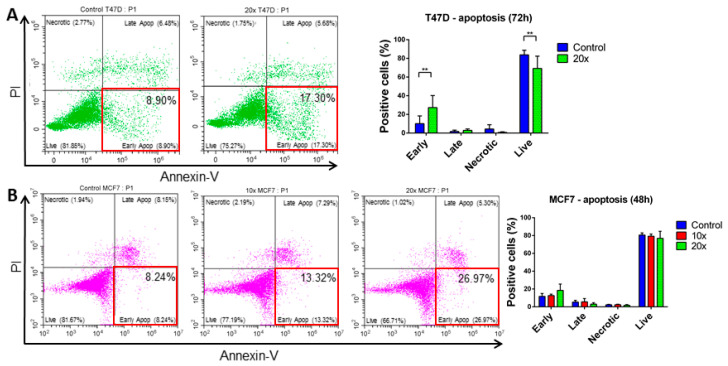
Apoptosis detection in T47D and MCF7 cells. Early apoptotic cells (Early; red square highlighted areas, lower right squares) of T47D were increased significantly (**A**) compared to controls after 72 h methyl-donor treatments, while MCF7 showed only a tendency of increase after 48h (**B**). Each bar represents the average percentage of positive cells in early apoptotic, late apoptotic, necrotic, and live cells area from at least 3 repeats ± SD. Statistical significance was plotted as **: *p* < 0.01. Late: late apoptotic cells; Necrotic: necrotic cells; Live: live cells. ×10 and ×20: concentrations of methyl-donors.

**Figure 4:**
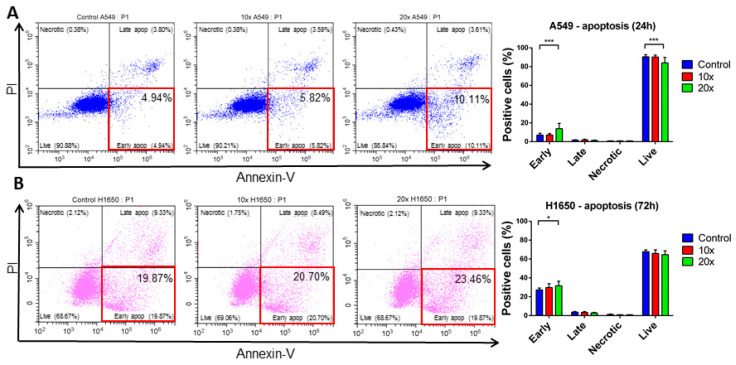
Apoptosis detection in A549 and H1650 cells by flow cytometry (**A**,**B**). Early apoptotic cells (red squared highlighted areas, lower right squares) were significantly elevated at the highest concentration of methyl-donor treated A549 and H1650 cell lines after 24 h (**A**) and 72 h (**B**), respectively, compared to control. Each bar represents the average percentage of positive cells in the early apoptotic, late apoptotic, necrotic, and live cells area from at least 3 repeats ± SD. Statistical significance was plotted as *: *p* < 0.05; ***: *p* < 0.001. Early: early apoptotic cells; Late: late apoptotic cells; Necrotic: necrotic cells; Live: live cells. ×10 and ×20 concentrations of methyl-donors.

A significantly elevated number of Annexin-V single positive, early apoptotic cells were detected in T47D breast cancer (*p* < 0.01) by flow cytometry after methyl-donor treatments compared to controls (Figure 3A), however only a tendency (*p* = 0.41) of increase was seen, and only at 48 h (Figure 3B), but not at 72 h in MCF7 cells. Moreover, significantly elevated early apoptotic cells were detected both in A549 and H1650 lung cancer cell lines (*p* < 0.001 and *p* < 0.05, respectively) (Figure 4A,B).

**Figure 5:**
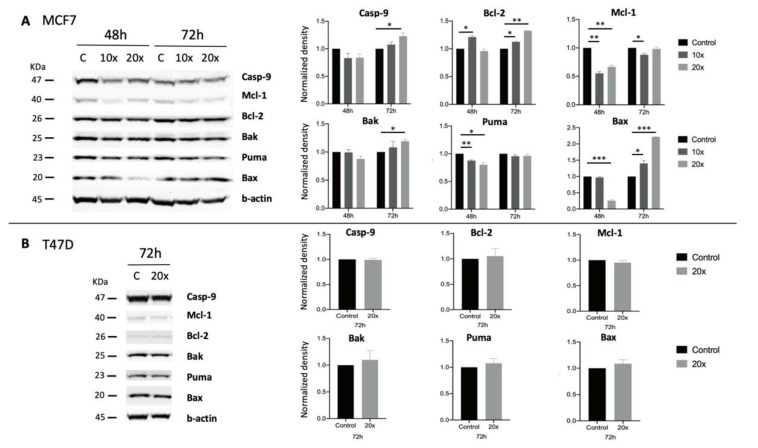
Western blot analysis of apoptotic proteins in methyl-donor treated MCF7 and T47D cell lines. Pro-apoptotic Caspase-9, Bak, and Bax were significantly increased in MCF7 cells after 72 h treatments, while the anti-apoptotic Mcl-1 significantly diminished. Interestingly, the pro-apoptotic Puma and the anti-apoptotic Bcl-2 changed significantly in an opposite way as expected (**A**). In the case of T47D, none of the changes were significant (**B**). Each bar represents the average normalized density from at least 3 repeats ± SD. Statistical significance is plotted as *: *p* < 0.033, **: *p* < 0.002, or ***: *p* < 0.001. C: control; Casp-9: Caspase-9. ×10 and ×20: concentrations of methyl-donors.

Furthermore, we investigated the methyl-donor-induced changes in the expression of pro- and anti-apoptotic proteins using Western blot. The dynamism of changes varied at different time points and methyl-donor concentrations. In MCF7 breast cancer cells, the intrinsic apoptotic pathway induced Caspase-9 and the pro-apoptotic Bak and Bax protein levels were significantly increased at 72 h (*p* < 0.033, *p* < 0.033, and *p* < 0.001, respectively), in line with the significant reduction of the anti-apoptotic Mcl-1 protein (*p* < 0.033). However, the anti-apoptotic Bcl-2 levels were elevated both after 48 h and 72 h (*p* < 0.033 and *p* < 0.002, respectively), and the pro-apoptotic Puma levels were decreased significantly (*p* < 0.033). These changes occurred mostly when the higher, i.e., ×20 concentration of methyl-donors were used (Figure 5A). In T47D lung cancer cells, neither the pro-apoptotic, nor the anti-apoptotic proteins changed significantly after treatment (Figure 5B).

**Figure 6:**
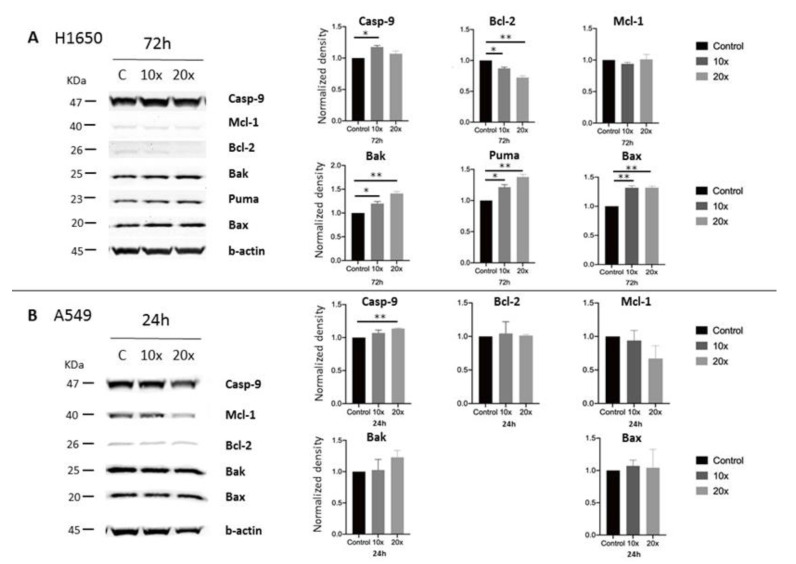
Western blot analysis of apoptotic proteins in methyl-donor treated A549 and H1650 cell lines. Clear opposite changes of pro-apoptotic vs. anti-apoptotic proteins were seen in both lung cancer cell lines. In H1650 cells, all protein changed significantly after the treatments, except the Mcl-1 (**A**). In A549, only the Caspase-9 increased significantly (**B**). Each bar represents the average normalized density from at least 3 repeats ± SD. Statistical significance is plotted as *: *p* < 0.033, **: *p* < 0.002. C: control; Casp-9: Caspase-9. ×10 and ×20: concentrations of methyl-donors.

In the H1650 lung cancer cell line, the pro-apoptotic Caspase-9, Bak, Puma, and Bax protein levels were elevated, while the anti-apoptotic Bcl-2 levels decreased significantly at unchanged Mcl-1 levels, after methyl-donor treatment. All significant changes were seen at both methyl-donor concentrations, except in the case of Caspase-9, where only the lower concentration resulted in significant increase (Figure 6A). In A549 cells, only the increase of Caspase-9 levels was significant (*p* < 0.002), but Bak and Mcl-1 also showed a strong tendency of increase and reduction, respectively (*p* = 0.10 and *p* = 0.15, respectively) (Figure 6B).

### 2.4. Investigating the MAPK/ERK and AKT Signaling Pathways

Then, we tested how methyl-donor treatments affect major growth and proliferation related pathways by focusing on Akt, and p-p44/42 MAPK (p-Erk1/2) expression. We also explored the activation and expression of p53 protein through detecting its forms phosphorylated either at Ser15 or Thr55. 

**Figure 7:**
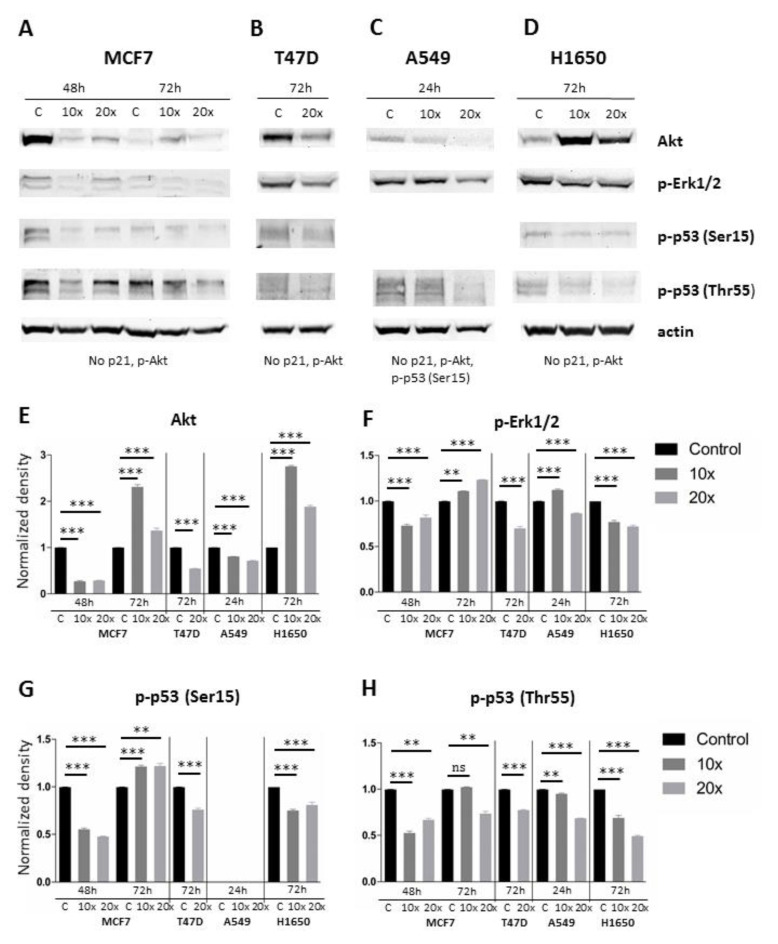
Changes of the level of selected signaling proteins after methyl-donor treatment in MCF7, T47D, A549, and H1650 cell lines. (**A**–**D**). Results of the Western-blot analysis of Akt, p-Erk1/2, p-p53(Ser15, p-p53(Thr55) and actin as a loading control. (**E**). Statistical analysis of Akt protein expression in all cell lines (*n* > 3). (**F**). Statistical analysis of p-Erk1/2 protein expression in all cell lines (*n* > 3). (**G**). Statistical analysis of p-p53(Ser15) protein expression in all cell lines (*n* > 3). (**H**). Statistical analysis of p-p53(Thr55) protein expression in all cell lines (*n* > 3). Akt and p-Erk1/2 decreased significantly in most cases. Pan Akt, however, increased significantly at 72 h in MCF7, and similarly in H1650 cells (**E**), as well as the p-Erk1/2 at 72 h in MCF7 and at ×10 dilution in A549 (**F**). Phosphorylation of p53 (Thr55) decreased significantly in all cases (**H**), while activated p53 (Ser15) increased significantly at 72 h in MCF7 (**G**). Each bar represents the average normalized density from at least 3 repeats ± SD. Statistical significance is plotted as **: *p* < 0.002, and ***: *p* < 0.001. C: control. ×10 and ×20: concentrations of methyl-donors.

Methyl-donor treatment significantly reduced Akt in MCF7 at 48 h, T47D and A549 (Figure 7A–E), as well as the p-Erk1/2 protein levels in MCF7 at 48h, T47D, and A549 at 20x concentration and in H1650 (Figure 7A–D,F) compared to the controls. However, pan Akt as well as the p-Erk1/2 increased at 72 h in MCF7 cells, and Akt in H1650 cells in the same manner as in MCF7. Additionally, p-Erk1/2 increased in A549 cells when using the lower treatment dilution (Figure 7F). Phosphorylation of p53 at Ser15 was significantly increased only in MCF7 cells after 72 h at both methyl-donor dilutions (Figure 7A–D,G). Activated p53 (Thr55) was significantly decreased in all cases as a result of the methyl-donor treatments (Figure 7A–D,H). Neither p-Akt, nor p21 expression could be detected in any cases, also as p53 (Ser15) in A549 cells.

## 3. Discussion

Several earlier studies have suggested that dietary methyl-donors could contribute to cancer prevention as high intake of methyl-donors and related vitamins could reduce the risk of breast and lung cancers [8,9,10,11]. Accumulating data have shown recently how dietary vitamins and herbal extracts can influence signaling pathways both in normal and tumor cells [32,33,34,35]. In this work, we further studied the effects of methyl-donors (using a mixture of L-methionine, choline chloride, folic acid, and vitamin B12) on breast (MCF7 and T47D) and lung (A549 and H1650) cancer cell lines. We could confirm our starting hypothesis by showing that methyl-donor treatment can reduce tumor cell proliferation rate and activate cell death-related pathways resulting in measurable programmed death response of cancer cells. Since methyl-donor treatment affected the proliferation rate in all tested cell lines, we focused on two main growth and proliferation pathways, MAPK/ERK and PI3K/AKT, which are most frequently involved in tumorigenesis [35]. Indeed, p-Erk1/2 protein levels were significantly decreased after the treatments. The MAPK/ERK pathway is known to phosphorylate and thus inactivate Caspase-9 and subsequently inhibit apoptosis [36]. This was further supported by the post-treatment downregulation of Akt levels. The PI3K/AKT pathway is also frequently upregulated in breast cancers and associated with metastatic growth, chemotherapy resistance, and poor prognosis [37]. *AKT1* overexpression has a pro-proliferative and anti-apoptotic effect [38,39], and its knockdown can revert these activities [40]. Hypoxia-induced *AKT2* can induce cancer proliferation [41], while downregulated *AKT2* is linked with cell cycle arrest and reduced proliferation [42,43]. Of the four cell lines, only at 72 h of MCF7 breast and H1650 lung cancer showed elevated Akt levels upon methyl-donor treatment, which, however, was not associated with elevated proliferation at the latter probably due to the compensation by the reduced p-Erk1/2 levels.

These were in line with the findings by another group showing significantly reduced proliferation in both MCF7 and T47D cells after methyl-donor treatment, who detected significantly decreased expression of Bcl-2 protein in T47D, but not the MCF7 cell lines [32].

Thus, we analyzed the apoptotic process, and additionally, the cell cycle changes, focusing on the SubG1 fractions, which contain the apoptotic cells as well. Although we detected a significantly increased subG1 fraction only in breast cancer cell lines, a similar tendency was seen also in the NSCLC cells. The Annexin-V positive cell fractions after treatments revealed that the number of the single positive, early apoptotic cells increased significantly in T47D, A549, and H1650 cell lines, except in MCF7 cells. 

In line with the elevated apoptosis, we tested the expression of the pro- and anti-apoptotic pathway proteins after methyl-donor treatment. We confirmed the increased expression of the pro-apoptotic proteins Bak and Bax, which are involved in mitochondrial membrane permeabilization [44]. This was in line with the significantly elevated level of Caspase-9 detected in cases (except T47D), which is activated in the apoptosomes upon mitochondrial membrane damage by the potentially released cytochrome C [36,44]. As a further support of apoptosis pathway activation, we found reduced anti-apoptotic Mcl-1 protein levels, which could have prevented mitochondrial membrane damage against Bak and Bax activity [45,46,47]. Although, none of these changes were significant in T47D cells, Annexin-V indicated apoptosis and elevated subG1 fraction was detected also in these cells. Surprisingly, in MCF7 cells the anti-apoptotic Bcl-2 levels increased after treatment, which (along with Bcl-xL) can inhibit Puma to mediate apoptosis resistance [48]. Indeed, we detected a significant decrease of Puma levels and lack of treatment induced an increase of early apoptotic cells in MCF7. As opposed to Mcl-1, we found the dose dependent significant reduction of Bcl-2 only in H1650 cells after the treatment.

Phosphorylation of p53 at Ser15 and Thr55 plays an important role in the nucleocytoplasmic shuttling of p53 [49]. Inhibition of p-p53 (Thr55) restores the nuclear localization of p53 and sensitizes to DNA damage [50,51], while DNA damage induces phosphorylation at Ser15 [52,53]. Therefore, the reduced p-p53 (Thr55) levels after methyl-donor treatment may restore functioning of p53 and allow subsequent activation of downstream pro-apoptotic signaling. Curcumin and vitamin E can downregulate p-p53 at Ser15 to protect against cytotoxicity caused by chemotherapeutic agents in normal lung epithelial cells [54]. We expected from nutrients to support normal cell functions and sensitize the tumor cells to subsequent targeted therapies, and therefore did not expect increased phosphorylation of p53 (Ser15) without chemotherapies or DNA-damaging agents. We can conclude that methyl-donor treatment may restore p53 nuclear localization and protect against unnecessary metabolic stress, while inducing apoptosis in cancer cells.

Vitamin C induces apoptosis, without affecting p53 [55], and mediates anti-proliferative effects as well in several drug resistant breast cancer cell lines [56]. Additionally, it has a synergistic effect with chemotherapies [57], without having a significant impact on normal cells [55]. Similarly, vitamin B2 sensitizes cancer cells to vitamin C-induced cell death [58]. Although, vitamin B1 (thiamine) did not induce apoptosis, but reduced cell viability selectively on cancer cells, with significant increase of the basal, maximum, and ATP production oxygen consumption in MCF7 cells, but not in MCF10A. Moreover, it reduced the extracellular lactate levels of both cancer and normal cell lines, and increased cellular pyruvate dehydrogenase (PDH) activity in breast cancer MCF7 cells [34]. When PDH activity is decreased, glycolysis replaces the failed aerobic metabolism, which results in an elevated lactate level [59,60]. Tumor cells prefer glycolysis for tumor growth, thus restoring the aerobic metabolism slows down the tumor growth [61]. Methyl-donors have only a moderate, non-significant effect on normal cell growth, similar to vitamin C [32,55].

An appropriate diet, rich in complex food intake, including ω-3 fatty acids, fruits, and vegetables can be used to counteract cancer-related fatigue (CRF), particularly in patients with breast cancers [62]. Although, the lack of standardization in dietary interventions and heterogeneity of study design, nutrition therapies, and quality of life measures may not allow the drawing of firm conclusions, preliminary data indicate that plant-based nutrition therapy can support CRF [63,64].

In conclusion, dietary methyl-donors may contribute to reducing MAPK/ERK and AKT pathways and protecting p53 functions, which may sensitize tumor cells to chemotherapy-induced DNA damage. Methyl-donors are also supposed to reduce cytotoxicity in normal cells and unnecessary metabolic stress in cancer cells to buffer the adverse effects of oncotherapies. Further studies are required to confirm the effectiveness of methyl-donors and to standardize their application in the clinical setup.

## 4. Materials and Methods

### 4.1. Cell Culture Conditions

Breast (MCF7, T47D) and lung cancer (A549, H1650) cell lines were purchased from ATCC (Manassas, VA, USA), and DSMZ (Braunschweig, Germany), and used for in vitro experiments. MCF7 human breast adenocarcinoma (HTB-22) and H1650 lung adenocarcinoma (CRL-5883) cell lines were cultured in RPMI1640 (LM-R1640; Biosera, Nuaille, France). A549 lung carcinoma (CCL-185) cell line was grown in Dulbecco’s modified Eagle medium (DMEM with 4.5 g/L glucose, BE12-604Q; Lonza, Basel, Switzerland). T47D human ductal carcinoma (HTB-133) cell line was maintained in Ham’s F12 Nutrient Mix (21765-029; Thermo Fisher Scientific, Waltham, MA, USA).

All media were supplemented with 10% fetal bovine serum (FB-1090; Biosera, Nuaille, France) and 0.4% gentamycin (Sandoz, Basel, Switzerland; 80 mg/2 mL). For T47D, 10 µg/mL insulin (12585-014; Thermo Fisher Scientific, Waltham, MA, USA) also was added to medium. All of the cell lines were kept under standard culture conditions (5% CO_2_, 37 °C).

All cell lines were regularly tested for *Mycoplasma* sp., applying a PCR test following the methodological article written by Uphoff et al. [65]. Only negative cell lines were used for research purposes.

### 4.2. Methyl-Donor Treatments

l-methionine, choline chloride, folic acid, and vitamin B12 were purchased from Sigma Aldrich (M5308, C7527, F8758, V6629, respectively; St. Louis, MO, USA). Cells were grown in culture media until 50% confluence, then were treated with different concentrations (×1, ×10, ×20) of the mixture of methyl-donors. Basal concentration (×1) was: 17 mg/L l-methionine, 9 mg/L choline chloride, 3 mg/L folic acid, and 2 mg/L vitamin B12. These concentrations were used according to a previous study of Park et al. [32].

### 4.3. Cell Proliferation Assay

Cells were plated onto a 96-well plate at a cell density of 1.5–3 × 10^4^ cells/mL depending on cell lines. When cells reached 50% confluence, culture media were changed to methyl-donor supplemented media and incubated for 24–72 h. Cell growth was measured at 24 h, 48 h, or 72 h by a colorimetric MTS (3-(4,5-dimethylthiazol-2-yl)-5-(3-carboxymethoxyphenyl)-2-(4-sulfophenyl)-2H-tetrazolium, inner salt) cell proliferation assay (CellTiter 96® AQueous One Solution Cell Proliferation Assay, G3582; Promega, Madison, WI, USA) according to the manufacturer’s instructions. Briefly, 20μL of the reagent was added into 100 μL of culture medium in each well at 24 h, 48 h, or 72 h of treatments. After 2 h of incubation, the absorbance of the soluble formazan product was measured at 490 and 690 nm with a plate reader (Labsystems Multiskan MS, Thermo Fisher Scientific Waltham, MA, USA).

### 4.4. Detection of Apoptosis

Cells were plated onto 6-well plates at a cell density of 3–5 × 10^4^ cells/mL depending on cell lines. After 24, 48, or 72 h treatments of methyl-donor, cells were washed with PBS then detached by trypsin-EDTA solution (XC-T1717; Biosera, Nuaille, France). After centrifugation (1500 rpm, 5 min), apoptotic cell fraction was determined by using FITC Annexin V Apoptosis Detection Kit with PI (640914; BioLegend, San Diego, CA, USA). Annexin V and/or PI positive cell fractions were detected by CytoFLEX flow cytometer using CytExpert software (Beckman Coulter, Indianapolis, IN, USA).

### 4.5. Cell Cycle Measurement

Tumor cell lines were plated and treated as described above in Section 4.4. After washing steps, cells were fixed in ice cold 70% ethanol at room temperature for 20 min, then were kept at −20 °C for an additional 30 min. Cells then were washed twice in PBS. After resuspension in PBS containing 1% RNaseA (R5503; Sigma Aldrich, 10 mg/mL), 20 µL propidium iodide solution was added (P3566, Thermo Fischer Scientific Inc, Waltham, MA, USA; 1 mg/mL) and the samples were incubated for 1 h at 4 °C. Cell cycles were detected by CytoFLEX flow cytometer using CytExpert software (Beckman Coulter, Indianapolis, IN, USA).

### 4.6. Western Blot

Cells were lysed in radioimmunoprecipitation assay (RIPA)-buffer, supplemented with 0.5 mM Na-orthovanadate, 10 mM NaF, and 1:200 Protease Inhibitor Cocktail (P8340, Sigma-Aldrich, St. Louis, MO, USA). Lysates were collected in tubes then centrifuged on 12,000 rpm for 15 min. Total protein concentration was determined using Pierce Rapid Gold BCA Protein Assay Kit (A53226; Thermo Fisher Scientific, Waltham, MA, USA). Cell extracts were mixed with ×5 sample loading buffer containing 2-mercaptoethanol (1610710; Bio-Rad, Hercules, CA) and heated to 95 °C for 5 min.

For Western blot analysis, 12–30 μg of total proteins were loaded and run in 10% sodium dodecyl sulphate polyacrylamide gel (SDS-PAGE) at 80 V for 20 min, then at 180 V for 50 min on Mini Protean vertical electrophoresis equipment (Bio-Rad, Hercules, CA, USA). Proteins were transferred onto Immobilon-P PVDF transfer membrane (IPVH00005; Merck KGaA, Darmstadt, Germany) by blotting at 100 V for 60 min at +4 °C. Membranes were blocked, then incubated with primary antibodies overnight at +4 °C (dilution in Table 1). β-actin was used for loading control. After washing steps, membranes were incubated with horseradish peroxidase (HRP)-labelled secondary antibodies (Table 1) for 60 min at room temperature. Membranes were detected using SuperSignal West Pico Chemiluminescent Substrate Kit (34080; Thermo Fisher Scientific, Waltham, MA, USA) and were visualized by iBright FL1500 Imaging System (Thermo Fisher Scientific, Waltham, MA, USA). Densitometric analysis of the immunoblots was performed using Image J software (developed by National Institute of Health (NIH) and Laboratory for Optical and Computational Instrumentation (LOCI), University of Wisconsin).

### 4.7. Statistical Analysis

All experiments were repeated at least n = 3 different times and are expressed as mean ± standard deviation (mean ± SD). We applied the non-parametrical t-test Wilcoxon matched-pairs signed rank test, one-way ANOVA, and two-way ANOVA with Bonferroni or Geisser–Greenhouse correction, and Dunnett’s test was used for multiple comparisons. We applied the GraphPad Prism software (GraphPad Software LLC, San Diego, CA, USA). The significance level was used as *: 0.01 < *p* < 0.05, **: 0.001 < *p* < 0.01, and ***: *p* < 0.001 in the case of proliferation, cell cycle experiments, and measurement of the level of apoptosis. For analyzing Western blots, the p value style of New England Journal of Medicine (NEJM) was used (*: 0.002 < *p* < 0.033, **: 0.001 < *p* < 0.002, and ***: *p* < 0.001).

## Figures and Tables

**Table 1:** Specifications and dilutions of the applied primary and secondary antibodies.

Name	Manufacturer	Cat. Number	Dilution	Host
Caspase-9	Cell Signaling	9502S	1:1000	rabbit
Bak (D4E4)	Cell Signaling	12105T	1:1000	rabbit
Puma	Cell Signaling	12450T	1:1000	rabbit
Bax (D2E11)	Cell Signaling	5023T	1:1000	rabbit
Bcl-2 (124)	Cell Signaling	15071S	1:1000	mouse
Mcl-1 (D5V5L)	Cell Signaling	39224S	1:1000	rabbit
p21 (SX118)	Santa Cruz Biotechnology	sc-53870	1:200	mouse
p-Erk1/2	Cell Signaling	4370S	1:2000	rabbit
p-p53 (B-3) (Thr55)	Santa Cruz Biotechnology	sc-377553	1:200	mouse
p-p53 (Ser15)	Cell Signaling	9284T	1:1000	rabbit
p-Akt	Cell Signaling	3787S	1:1000	rabbit
Akt (pan) (11E7)	Cell Signaling	4685S	1:1000	rabbit
beta-Actin (13E5)	Cell Signaling	4970S	1:5000	rabbit
Anti-mouse IgG, HRP-linked	Cell Signaling	7076S	1:1000	horse
Anti-rabbit IgG, HRP-linked	Cell Signaling	7074S	1:1000	goat
